# Organic Psychosis Secondary to Untreated Graves’ Disease: A Case Report

**DOI:** 10.7759/cureus.108994

**Published:** 2026-05-16

**Authors:** Khairul Arif Ahmad, Mohammad Che Man

**Affiliations:** 1 Family Medicine, International Islamic University Malaysia, Kuantan, MYS

**Keywords:** acute psychosis, adjunct lithium therapy, graves’ disease, hyperthyroidism, neuropsychiatric manifestation, thyrotoxicosis

## Abstract

Hyperthyroidism is characterized by elevated circulating thyroid hormone levels with suppressed levels of thyroid-stimulating hormone (TSH). Common manifestations include weight loss, tremor, palpitations, and heat intolerance, while psychosis is a rare neuropsychiatric manifestation. We report the case of a 47-year-old woman who presented with a three-month history of worsening irritability, destructive behavior, irrelevant speech, and multiple delusions, including grandiose, persecutory, and erotomanic types. She also exhibited excessive spending behavior, increased energy, and a reduced need for sleep. Further history revealed symptoms suggestive of untreated hyperthyroidism for three years before presentation. Physical examination showed exophthalmos, goiter, tachycardia, and mild hypertension. Thyroid function tests confirmed thyrotoxicosis, and she was diagnosed with organic psychosis secondary to Graves' disease. Despite treatment with antithyroid medication and beta-blockers, thyrotoxicosis and psychotic symptoms initially persisted. Adjunctive treatment with lithium, combined with psychotropic medication, resulted in marked biochemical and clinical improvement. This report highlights psychosis as a rare manifestation of untreated hyperthyroidism and emphasizes the importance of early recognition and multidisciplinary management involving both endocrinology and psychiatry.

## Introduction

Thyroid hormones, namely triiodothyronine (T3) and thyroxine (T4), regulate body metabolism and are important for normal growth, brain development, and cardiovascular, nervous, and gastrointestinal function [1,2]. Their secretion is controlled by the hypothalamic-pituitary-thyroid axis through a negative feedback mechanism involving thyrotropin-releasing hormone (TRH) and thyroid-stimulating hormone (TSH) [2].

Hyperthyroidism is defined as a biochemical state characterized by elevated thyroid hormone levels with suppressed levels of TSH [1]. This occurs due to negative feedback inhibition from increased circulating thyroid hormones on the hypothalamus and anterior pituitary, leading to reduced TSH secretion [3]. The most common causes of hyperthyroidism include Graves’ disease, toxic multinodular goiter, thyroiditis, and toxic thyroid adenoma [1,3]. Patients with hyperthyroidism typically present with weight loss, heat intolerance, tremor, palpitations, increased bowel movements, and anxiety, while neuropsychiatric manifestations are more likely in severe cases.

Globally, hyperthyroidism affects approximately 2% of the population, with a female predominance up to 10 times that of males [3]. Among these patients, psychosis is a rare manifestation, occurring in approximately 1% of cases [4,5]. This case report highlights a patient with untreated hyperthyroidism who presented with psychosis, the combined therapeutic approach employed in management, and the resolution of symptoms following normalization of thyroid function.

## Case presentation

A 47-year-old woman presented with a three-month history of worsening irritability, destructive behavior, and irrelevant or disorganized speech. She exhibited multiple delusions, including grandiose, persecutory, and erotomanic types. She also demonstrated excessive spending behavior, notably through frequent donations, increased energy levels, and a reduced need for sleep. She did not report auditory or visual hallucinations, nor any depressive symptoms. She had no prior history of similar episodes. These behavioral changes were noticed by family members, as she had previously been described as a quiet and timid person. Further history revealed associated symptoms of palpitations, heat intolerance, weight loss, hair loss, and hand tremors over the past three years, for which she had not sought medical attention. She had no significant past medical history, no family history of thyroid disease or psychiatric illness, and no history of substance abuse.

**Figure 1:**
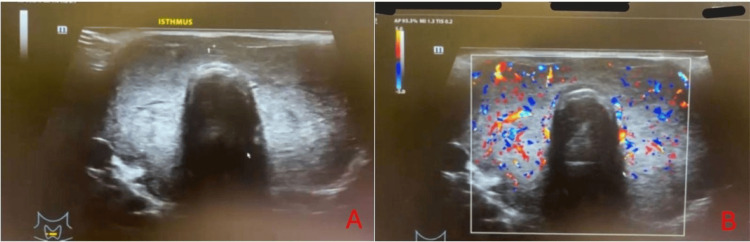
Ultrasound of the thyroid gland showing features of diffuse goiter (A) Transverse grayscale image demonstrating diffuse enlargement of the thyroid gland with heterogeneous echotexture involving the thyroid isthmus. (B) Color Doppler image demonstrating increased diffuse vascularity within the thyroid parenchyma, consistent with Graves’ disease

On presentation to a primary care clinic, she was agitated and exhibiting psychotic behavior. Physical examination revealed exophthalmos and goiter. She was afebrile, with mild hypertension and a tachycardic heart rate. She was referred to a tertiary center for inpatient management of psychosis with suspected underlying hyperthyroidism. Initial investigations confirmed thyrotoxicosis, with elevated free T4 (67 pmol/L) and suppressed TSH (<0.005 uIU/mL). Anti-thyroid peroxidase (anti-TPO) antibodies were elevated. Neck ultrasound revealed a goiter with features suggestive of thyroiditis, as illustrated in Figure 1.

The patient was diagnosed with organic psychosis secondary to Graves’ disease and was co-managed by the psychiatric and endocrine teams. Her clinical course was complicated by persistent thyrotoxicosis, poor insight, and non-adherence to treatment, resulting in multiple hospital admissions before stabilization of both psychiatric symptoms and thyroid hormone levels. She was treated with antithyroid medications, beta-blockers, and adjunctive lithium to achieve biochemical control. Antipsychotic therapy was also initiated to manage acute psychotic symptoms.

A summary of the patient’s clinical progression, management, and outcomes is shown in Table 1. Her condition improved significantly following the normalization of thyroid function. Table 2 summarizes her laboratory results throughout the clinical course. On the latest psychiatric review, she demonstrated complete resolution of psychotic symptoms and had returned to her baseline level of functioning. She achieved a euthyroid state on antithyroid therapy and remains on regular follow-up with the endocrine team.

**Table 1:** Chronological progression of clinical features, management, and outcomes The table presents the chronological progression of the patient’s clinical features, biochemical parameters, and treatment adjustments, highlighting the role of lithium as an adjunct in achieving both psychiatric and biochemical stabilization

Admission	Clinical presentations and lab results	Management	Outcome
First admission	Irritable, talkative, poor sleep, thyrotoxic state with positive autoimmune markers	Started carbimazole 20 mg OD, propranolol 20 mg OD	Calm and cooperative; persistent persecutory delusion and grandiosity
Second admission (defaulted medication)	Irrelevant speech, irritability, grandiosity, destructive behavior with persistent thyrotoxicosis	Added lithium 300 mg OM/600 mg ON, increased carbimazole dose to 20 mg BD, increased propranolol to 40 mg BD, added quetiapine 300 mg BD, and PRN clonazepam	Calm, persistent delusion; poor insight
Third admission	Reduced need for sleep, disorganized behavior; FT4 showed reduction, but borderline high therapeutic lithium level	Reduced lithium to 300 mg BD and carbimazole to 10 mg BD, propranolol was continued, adjusted quetiapine to 300 mg OM/400 mg ON	Improved sleep, better compliance, and reduced psychotic symptoms
Follow-up	Clinically stable with near normalization of thyroid function and therapeutic lithium level	Continued maintenance therapy	Marked clinical and biochemical improvement

**Table 2:** Patient’s laboratory parameters at presentation and during follow-up Persistent thyrotoxicosis was observed, with a reduction in thyroid hormone levels following adjunct lithium initiation during the second admission. Positive autoimmune markers favored Graves' disease as the underlying etiology and suggested relapse risk TSH: thyroid-stimulating hormone; T4: thyroxine; TPO: thyroid peroxidase

Parameter	Patient value				Reference range
	First admission	Second admission	Third admission	Follow-up	
TSH, uIU/mL	<0.005	<0.005	<0.005	<0.005	0.38–5.33
Free T4, pmol/L	66.9	73	3.4	10	7.9-14.4
Anti-TPO, IU/mL	337	-	-	-	<34
Lithium level, mmol/L	-	-	1.2	0.79	0.6-1.2
Anti-TSH receptor, IU/L	-	-	-	22.30	<1.75

## Discussion

The exact mechanism underlying psychosis in hyperthyroidism remains poorly understood. Several theories have been proposed, including increased β-adrenergic receptor activity and a hyperadrenergic state induced by excess thyroid hormone, leading to heightened dopaminergic and noradrenergic activity in the brain [6-8]. In addition, thyroid hormone excess may disrupt neurotransmitter regulation within the limbic system, particularly affecting dopamine and serotonin pathways, resulting in psychiatric manifestations such as delusions, hallucinations, and mood disturbances [4,5,9]. Autoimmune and inflammatory processes affecting the central nervous system have also been suggested as contributing factors to these neuropsychiatric symptoms [7].

Although psychosis is an uncommon manifestation of hyperthyroidism, several cases have been reported in the literature. Previous reports describe patients with Graves disease presenting with psychotic symptoms, including aggression, paranoia, hallucinations, and disorganized behavior, which improved following normalization of thyroid hormone levels with antithyroid treatment [4,5,10]. In addition, Sumi et al. demonstrated a temporal association between thyrotoxicosis and worsening psychotic manifestations, including the emergence of visual hallucinations during the thyrotoxic state [11]. These findings further support the link between thyroid hormone excess and neuropsychiatric disturbances.

Previous literature suggests that psychosis is more likely to occur in patients with severe or untreated thyrotoxicosis, including those with markedly elevated thyroid hormone levels or thyroid storm [8,11]. Delayed treatment may further increase this risk, and psychotic symptoms can occasionally persist despite definitive therapy [12]. Psychosis has been reported more commonly in autoimmune hyperthyroidism, particularly Graves' disease and thyroiditis [4,5,9,11]. A recent review by Taha et al. also found that most reported cases involved female patients [13]. In addition, thyrotoxicosis may worsen psychiatric manifestations in individuals with underlying mental health disorders such as schizophrenia or bipolar disorder [11]. In this case, several factors potentially associated with the development of psychosis were identified, including longstanding untreated hyperthyroidism, persistent biochemical thyrotoxicosis, and clinical findings suggestive of Graves' disease, an autoimmune etiology.

The primary goal of management in such cases is the normalization of thyroid hormone levels [4,14]. This can be achieved through antithyroid medications, radioactive iodine therapy, or surgery. Treatment should be individualized based on factors such as patient age, comorbidities, underlying etiology, likelihood of remission, and patient preference [14]. Beta-blockers are beneficial as they can cross the blood-brain barrier, providing both central and peripheral adrenergic blockade [4,5,14].

Lithium also has significant effects on thyroid function. It inhibits the release of thyroid hormones and reduces the peripheral conversion of T4 to T3, thereby lowering circulating hormone levels. It may be used as an adjunct in severe hyperthyroidism or as an alternative when standard antithyroid drugs are contraindicated [15,16]. As psychosis in this context is secondary to an underlying endocrine disorder, psychotropic medications are not always required. However, in patients with severe symptoms or poor insight, short-term use may be necessary until clinical stabilization is achieved [5].

## Conclusions

Hyperthyroidism should be considered in patients presenting with psychosis, as thyrotoxicosis represents a rare but reversible cause of neuropsychiatric manifestations. Available case reports suggest that psychosis may be associated with severe or untreated thyrotoxicosis, particularly in female patients and those with autoimmune etiologies or underlying psychiatric disorders. Early treatment with antithyroid drugs and beta-blockers is essential, while psychotropic medications and adjunctive therapies such as lithium may be considered based on the severity of clinical presentation. This report highlights the importance of prompt recognition and a multidisciplinary endocrine-psychiatric approach to optimize patient outcomes.
